# Dissociating the capture of attention from saccade activation by subliminal abrupt onsets

**DOI:** 10.1007/s00221-017-5040-2

**Published:** 2017-07-28

**Authors:** Tobias Schoeberl, Ulrich Ansorge

**Affiliations:** Department of Psychology, University of Vienna, Liebiggasse 5, 1010 Vienna, Austria

**Keywords:** Attentional capture, Subliminal attention, Anti-saccades

## Abstract

Attentional capture and effects on saccade metrics by subliminal abrupt onset cues have been studied with peripheral cues at one out of several (two to four) display locations, swiftly followed by additional onsets at the other display locations. The lead time of the cue was too short to be seen. Here, we were interested in whether such subliminal onset cues influenced saccades primarily by way of attention or by way of direct saccade activation. In separate blocks, participants made speeded pro-saccades towards a black target or anti-saccades away from the target. Prior to the targets, an abrupt onset cue was presented either at the same side as the target (valid condition) or at the opposite side (invalid condition). If cues influenced performance by way of attentional capture, we expected facilitation of target processing in valid compared to invalid conditions (cueing effect) in the pro- as well as in the anti-saccade task. If the cues activated saccades in their direction, we expected the cueing effect to drop in the anti-saccade task compared to the pro-saccade task because in the anti-saccade task the invalid cue would activate the finally required response, whereas the valid cue would activate the alternative response, leading to interference. Results were in line with the former of these possibilities suggesting that subliminal abrupt onsets influenced saccades by way of attention with no or little direct activation of saccades.

## Introduction

Visual attention can be driven endogenously or exogenously. Endogenous attention is top-down controlled attention (cf. Posner 1980). This type of attention is under strategic control and can be willingly directed to specific features, locations, or objects. Endogenous attention is, therefore, an indispensable prerequisite for the accomplishment of goals in complex tasks. Exogenous attention, by contrast, is stimulus-driven attention and is thought to emerge as a result of the physical salience of the impinging stimuli (Mulckhuyse and Theeuwes 2010; Theeuwes 1992, 2010). That is, certain stimuli are thought to be so salient that they automatically capture attention, regardless of the current goals, intentions, tasks, or top-down settings. Especially, new objects in a visual scene (i.e., abrupt onsets) are believed to have great potential to capture attention in an exogenous fashion (Theeuwes 1992, 2010; Yantis and Jonides 1990). Whether this is always true is subject of current debates because a number of studies with clearly visible, supraliminal abrupt onset cues of which participants were aware suggested that the capture of attention by abrupt onsets is under certain conditions contingent on top-down search settings (Folk et al. 1992; Goller et al. 2016). But at least when people are unaware of the abrupt onsets—that is, when the abrupt onsets are subliminal—abrupt onsets seem to capture attention in a truly exogenous fashion (Fuchs et al. 2013; McCormick 1997; Mulckhuyse et al. 2007). Researchers who studied exogenous capture of attention by subliminal abrupt onsets presented the onsets as peripheral attentional cues (McCormick 1997; Mulckhuyse et al. 2007; see also Posner and Cohen 1984). Peripheral cues are typically presented to the left or to the right of central fixation. After a variable delay, a target appears, and, if cues capture attention, searching for targets at the cue’s location (valid conditions) is facilitated compared to targets at the opposite side of the cue (invalid conditions). If the cue attracts attention to its location, targets that appear at the cue’s location will already be in the attentional focus and, therefore, these targets will be more readily represented and processed. Mulckhuyse et al. (2007) established a version of this cueing paradigm to study attentional capture by subliminal abrupt onset cues: Their abrupt onset cue was always presented to the left or to the right and preceded two additional onsets that looked exactly like the cue, one at fixation and one at the opposite side of the search display. The lead time of the cue was very short (16 ms). With this kind protocol, the participants subjectively perceived the three onsets to occur at the same time and the lead time of the cue remained unseen. Despite its invisibility, the abrupt onset cue captured attention as was evidenced by faster responses in valid conditions, when the cue was at the target location, compared to invalid conditions, when the cue was at the opposite side of the target (cueing effect). Subsequent studies “masked” their abrupt onset cues in the same way and confirmed this finding (Fuchs and Ansorge 2012; Fuchs et al. 2013; Schoeberl et al. 2015). In support of the assumption that the cueing effect by the subliminal abrupt onsets was due to exogenous attentional capture, the subliminal cueing effect was found when the cue was not predictive for the most likely target position (Fuchs and Ansorge 2012) and when the cue did not have any of the searched-for target-defining features (Fuchs et al. 2013; Schoeberl et al. 2015).

**Fig. 1:**
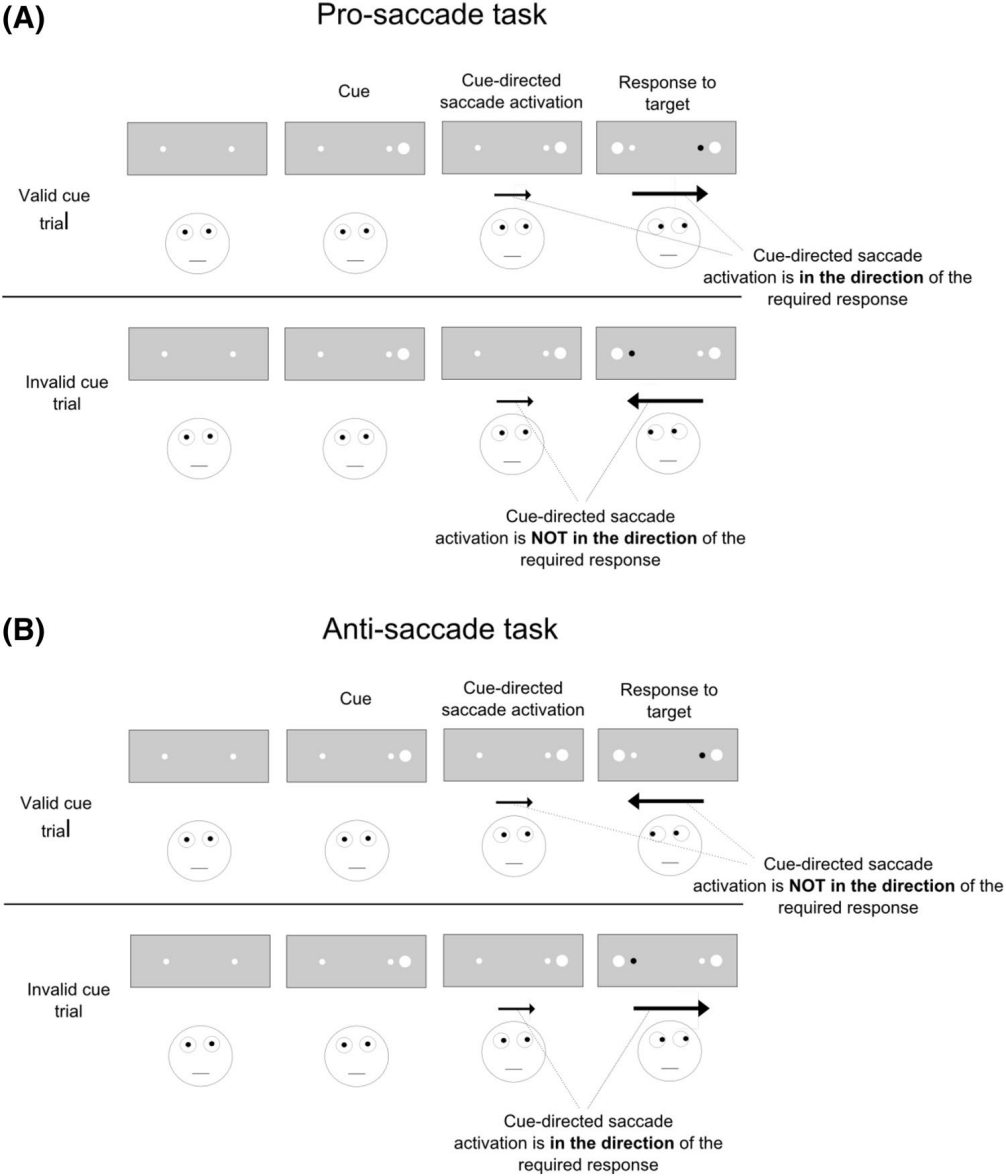
The figure illustrates the sequence of events and the differences between the pro- and the anti-saccade task with respect to the general rationale of the present study. **a** In the pro-saccade task (*upper part*), participants executed saccades towards a black target which appeared equally likely to the left or to the right. Valid cues (cues at the same side as the target) should activate saccades in the direction of the required response. Invalid cues (cues at the opposite side of the target) should activate a saccade in the opposite direction of the required response. Therefore, cue-directed saccade activation should foster cueing effects (facilitated responding in valid as compared with invalid conditions). **b** In the anti-saccade task, participants had to execute a saccade in the opposite direction of the target. Therefore, valid cues activated saccades in the opposite direction of the response saccade. Only invalid cues would activate a saccade in the direction of the response saccade with anti-saccades. As this should facilitate responding in invalid conditions compared to valid conditions, cueing effects were expected to be smaller with the anti-saccade task compared with pro-saccades if cues activated saccades. Only if cues did not activate saccades, but only captured attention so that targets in valid conditions were more readily processed compared with invalid conditions, the same cueing effects should emerge in the pro- as well as in the anti-saccade task

Here, we investigated if and to which extent subliminal abrupt onsets would also activate saccades towards the cue’s location. If subliminal abrupt onsets capture attention in an exogenous way as prior research suggested (Fuchs et al. 2013; Mulckhuyse et al. 2007; Schoeberl et al. 2015), saccade activation by the cues would be predicted under the assumption that exogenous attention and saccade activation are coupled (Belopolsky and Theeuwes 2012; Smith and Schenk 2012). The idea of the present study was to directly compare cueing effects by subliminal abrupt onsets in a pro-saccade task with the cueing effects in an anti-saccade task (Hallet 1978; Hallet and Adams 1980). In the pro-saccade task, participants have to shift their eyes towards a target. In the anti-saccade task, participants have to shift their eyes to the opposite side of a target. This difference allowed for different predictions depending on whether or not cue-directed saccade activation contributed to the subliminal cueing effect. In both tasks, attentional capture by the cues should lead to a cueing effect with facilitated responses in valid compared to invalid cue conditions as in prior studies (e.g., Mulckhuyse et al. 2007; Schoeberl et al. 2015). If cue-directed saccade activation contributed to the effects, the cueing effects should be moderated by the task (pro- or anti-saccade task) because cue-directed saccade activation should interfere with the required response in different ways: In the pro-saccade task, participants had to execute a saccade towards the target. Valid cues at the target location should, therefore, facilitate responding relative to cues away from the target, if the cues activated cue-directed saccades, not only because valid cues captured attention to the target location but also because valid cues activated the correct target-directed saccade. Invalid cues presented at the opposite side of the target, by contrast, would not activate the correct saccade when pro-saccades are required. In fact, if invalid cues activated saccades, they should activate the saccade into the wrong direction which should interfere with the target-directed saccade required for the correct response. Hence, both attentional capture by the cue as well as cue-directed saccade activation should foster the cueing effects in the pro-saccade task. In the anti-saccade task where participants had to execute a saccade to the opposite side of the target, however, cue-directed saccade activation should lead to a drop of the cueing effects because responding in invalid conditions should be facilitated compared to valid conditions by cue-directed saccade activation. Namely, cue-directed saccade activation by valid cues which are presented at the target location should interfere with the correct saccade because the correct saccade is not directed towards the target location where the cue is located but to the opposite side. Likewise, invalid cues presented at the opposite side of the target should facilitate responding if they elicited cue-directed saccades because cue-directed saccades would in this case be directed towards the same location as the required response. The cueing effect was, therefore, expected to drop with the anti-saccade task compared to the pro-saccade task if cue-directed saccade activation played a role. For an illustration of this rationale, see Fig. 1.

**Fig. 2:**
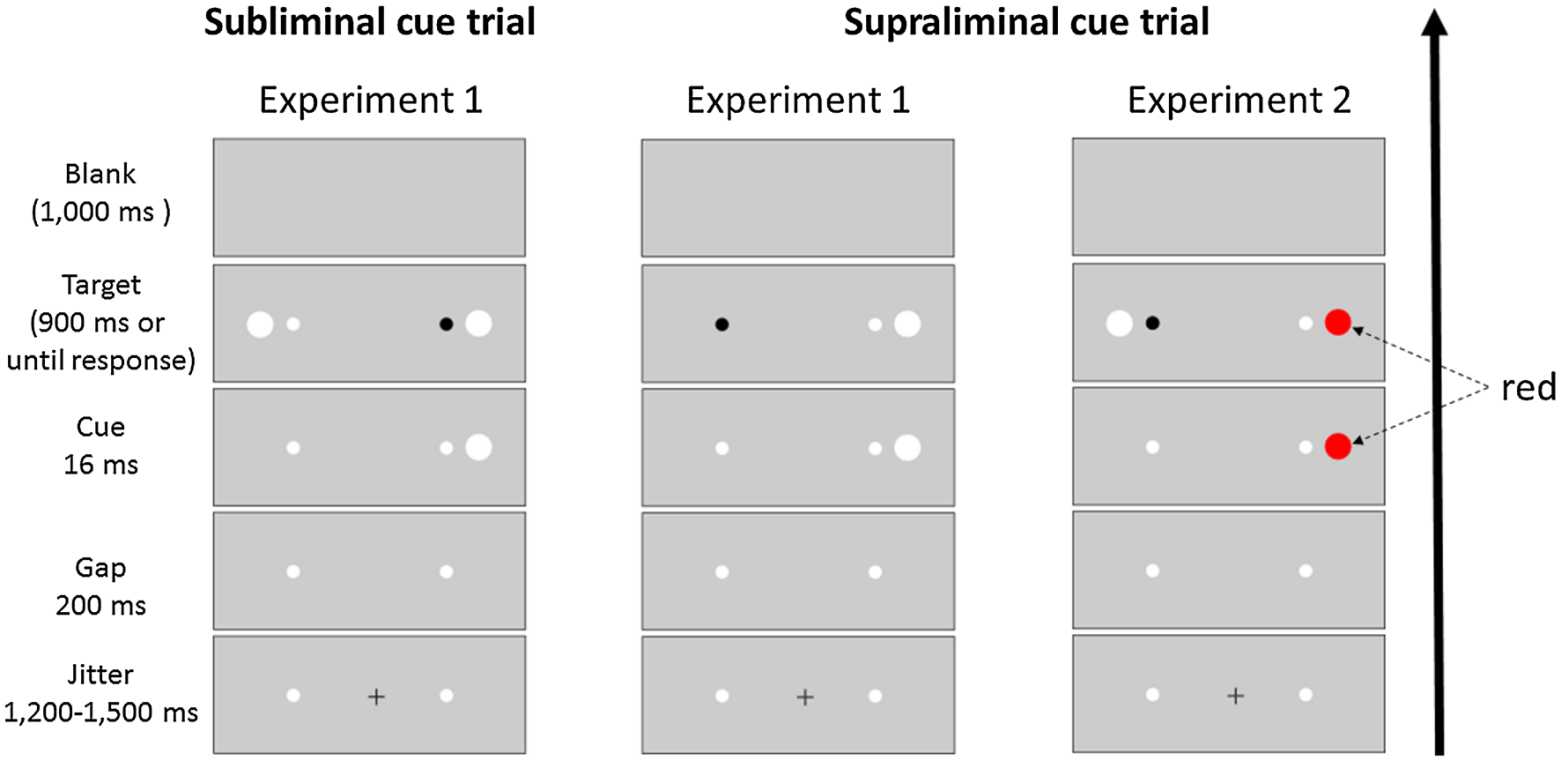
Depicted are sequences of events in subliminal and supraliminal cueing trials of *Experiments 1* and *2*. The *arrow on the left* indicates the flow of time from the *bottom* to the *top*. At the beginning of each trial, two white placeholders and a fixation cross were presented for 1200–1500 ms. After this, the fixation cross was turned off and, after a gap of 200 ms, the cue was presented. The cue was *white* in the subliminal cue trials and in the supraliminal cue trials of *Experiment 1*. It was* red* in *Experiment 2*. The cue was presented for 16 ms and then the target display appeared with the black target. In the subliminal cue trials of *Experiments 1* and *2*, a *white disc* appeared at the opposite side of the cue. The target display was presented for 900 ms or until a response or an erroneous saccade was detected. After this, a *blank display* was presented for 1 s before the next trial started (colour figure online)

To date, no study directly compared attention capture and saccade activation by subliminal abrupt onsets under similar conditions. Most studies investigated the capture of attention by subliminal abrupt onset cues by use of manual reaction time and accuracy as dependent variables, and these studies are, therefore, silent regarding the possibility of cue-directed saccade activation. There have been a few studies that masked the cues in similar ways and investigated the effects of subliminal abrupt onsets on saccade metrics (Mulckhuyse and Theeuwes 2010; Van der Stigchel et al. 2009; Weichselbaum et al. 2014), but these studies did not assess the capture of visual attention in a saccade-independent way. Van der Stigchel et al. (2009; see also Weichselbaum et al. 2014), for instance, presented subliminal abrupt onsets to the left and to the right of a vertical axis along which a saccade to a target as a response had to be executed. It was observed that this affected the saccade trajectories and saccade endpoints, but it is not certain how these effects related to the attention capturing potential of the onsets because the onsets were presented too far away from the targets (in each condition at least approximately 5°) as to benefit target processing by attracting attention to the target’s location. In one study Mulckhuyse and Theeuwes (2010) employed an oculomotor task that was comparable to their earlier study in which they measured the cueing effect of subliminal abrupt onset cues by manual responses (Mulckhuyse et al. 2007). In this oculomotor task, two placeholders were presented, one to the left and one to the right of fixation. One of these placeholders darkened in each trial. The darkened placeholder was the target and the participants had to execute a speeded pro-saccade towards the target once it had appeared. When the target appeared, the placeholders were flanked by two white disks nearby. One of these disks served as the cue and had its onset 16 ms prior to the onset of the second white disk and the target (Fig. 2, sequence of events on the left, shows the succession of events in the present study, which was almost identical to that in the study by Mulckhuyse and Theeuwes 2010). Like in prior studies with manual responses, the cue’s earlier onset was invisible because it had a lead time that was too short to be noticed. Mulckhuyse and Theeuwes confirmed in this study their findings with manual responses in that the subliminal cues facilitated target-directed saccades in valid compared to invalid conditions with a short cue-target interval. It remains unclear, however, whether this reflected attention or whether it reflected the activation of a saccade towards the location of the abrupt onset. With a pro-saccade task, the target and the goal of the required response are the same. That is, attentional capture by the valid cue, with the resulting facilitation of target processing at the cue’s location, would lead to a cueing effect (facilitation relative to invalid cues), and so would also the cue-elicited activation of a saccade because the valid cue appears at the goal location of the required response saccade (cf. Laidlaw et al. 2016; Weber et al. 1998). Here, we studied under similar conditions how attentional capture by the subliminal abrupt onsets cues related to saccade activation: We adapted the experimental setup of Mulckhuyse and Theeuwes (2010) and compared the performance with anti-saccades and with pro-saccades as described above.

## Experiment 1

In Experiment 1, we studied the cueing effect by subliminal cues with pro-saccades and with anti-saccades. In addition to the subliminal cue condition, which was almost identical to that in the study of Mulckhuyse and Theeuwes (2010), we employed a condition in which the cue was not followed by a second onset at the opposite side (see Fig. 2, sequence of events in the middle). As the cue was clearly visible in this condition, we will refer to this condition as the supraliminal cue condition. We included the supraliminal condition in order to show that abrupt onsets can be effective in eliciting cue-directed saccades. It is known that abrupt onsets have strong effects on saccade programming under similar conditions (e.g., McSorley et al. 2005, 2006; Van der Stigchel et al. 2009; van Zoest et al. 2008; Walker et al. 1997). A well-known example is the remote distractor effect: Abrupt onsets which are presented outside a limited area around the saccade goal impede goal-directed saccades compared to abrupt onsets that appear closer to the saccade goal (Walker et al. 1997). We, therefore, expected a drop or a reversal of the cueing effect in the anti-saccade task compared to the pro-saccade task at least with supraliminal cues because the supraliminal abrupt onsets were expected to interfere with saccade programming. We expected the results of the subliminal cues to mirror this pattern if they also led to saccade activation. If the subliminal cues only captured attention, we expected to see regular cueing effects in both tasks with subliminal cues.

We also introduced catch trials (20% of trials) in which the target was left out and participants had to withhold a response and keep fixation within a gaze contingent zone throughout the trial. Apart from increasing the incentive to search for the target because the correct response required detecting and locating the target, it allowed for an additional indirect assessment of the participants’ cue-directed saccade activations that was independent of the cueing effect itself: In catch trials, there was a cue but no target and participants were required to withhold a response and keep the eye gaze at the display center throughout the trial. If they failed to do so, the corresponding trial was counted as an error trial. The number of errors in the catch trials, therefore, served as a proxy for the participant’s response criterion and the degree to which the cue activated saccades. It is plausible to assume that a less conservative response criterion or strong saccade activation by the cue should lead to a higher error rate in the catch trials. We correlated the error rate in the catch trials (trials in which participants could not withhold a saccade) with the cueing effect in the pro- and anti-saccade conditions to test if saccade activation was indeed a likely underlying origin of the cueing effect. To the degree that saccade activation was responsible for the cueing effects (calculated as invalid reaction time minus valid reaction time), we expected to see positive correlations in the pro-saccade task and negative correlations in the anti-saccade task. This was expected for the subliminal as well as for the supraliminal cues to the extent that the cueing effects depended on saccade activation. No correlations were expected in the subliminal cue conditions if subliminal cues only captured attention.

### Methods

#### Participants

Twenty-four participants took part (*M*_Age_ = 21.63 years, SD = 2.7 years, 22 females), mainly in return for course credit or less often for a small monetary reward. Here and in Experiments 2 and 3, all participants had normal or corrected to normal vision and normal color vision as assessed by Ishihara color plates. Informed consent was obtained from each participant at the beginning of each experiment.

#### Apparatus, stimuli, and sequence of events in a trial

The experiment was conducted on a 12-inch CRT monitor (resolution 1024 × 768 pixels, refresh rate 60 Hz). Experiment builder software was used to control the presentation of the stimuli. Eye movements were recorded with a video-based eyetracker (EyeLink 1000, SR Research Ltd. Canada) from the dominant eye. The experimental design was adopted from Mulckhuyse and Theeuwes (2010). Participants viewed all stimuli binocularly on a gray background (6 cd/m^2^). Each trial started with a fixation cross at display center and a fixation check (i.e., a trial only started when a fixation occurred inside of a 1° visual angle squared area around the center of the display). The system was recalibrated if necessary. After a correct fixation, at the beginning of the trial, two white position placeholders of 0.4° diameter circles (14 cd/m^2^) appeared, one to the left and the other one to the right of the fixation point, both at an eccentricity of 6.5°. Following a random jitter between 1200 ms and 1500 ms, the fixation cross disappeared. After a gap of 200 ms, the cue, a 1.4° diameter white disk (14 cd/m^2^), was presented (cf. Mulckhuyse et al. 2007; Mulckhuyse and Theeuwes 2010) at 8° to the left or to the right of fixation (with a distance of 1.5° between the cue and the white placeholder). This first white disk was the cue. Sixteen milliseconds after cue onset, one of the white placeholders turned black. This was the target, a 0.4° black circle (<2 cd/m^2^). Participants were instructed to look for this black target that appeared inside only one of the placeholders. It had the same size as the white placeholder. In the subliminal condition, a second white onset that had the same luminance and size as the cue appeared at the same time as the target at the opposite side of the cue (at 8° eccentricity). In this way, the onset of the cue and the second white onset appeared to be simultaneous, and participants could not perceive that the cue appeared earlier. In the supraliminal cue condition, the second onset was left out so that it was easy to see that there was an abrupt onset on one side only (cf. Fuchs et al. 2013). In both, the subliminal and the supraliminal condition, the cue was not predictive for the upcoming target location. It appeared equally likely at the same side as the target (valid condition) or at the opposite side of the target (invalid condition). The target display was presented for 900 ms or until a response or an erroneous saccade was detected. That is, the two placeholders, one of which contained the black target, the cue and the onset at the opposite side remained on the display until a response was detected or until the time interval in which a valid response could be made was over. After this, a blank display was presented for 1 s before the next trial started. If the trial was interrupted at any point due to an error or because participants did not maintain fixation inside of the gaze contingent region before the target appeared, participants received written feedback that they had made an error and a blank display was presented for 1 s. For an overview of the sequence of events in a trial, see Fig. 2.

#### Task

Participants were instructed to make a speeded saccade either towards (pro-saccade task) the black target or to the white placeholder on the opposite side of the target (anti-saccade task). They were also instructed that either one or two white disks (the onset of cue and the onset at the opposite side) would be presented in addition to the target. Participants were told that these onsets were not relevant but they were not informed about the lead time of the cue in the subliminal cue trials. In each trial, a correct saccade was registered if a saccade was detected (velocity threshold 30°/s, acceleration threshold: 8000°/s/s) with a subsequent fixation within a 3° × 3° area around the target (in the pro-saccade task) or around the placeholder on the opposite side of the target (in the anti-saccade task). Once a correct response was detected, the target display was turned off and a blank display was presented for 1 s until the next trial started. Trials in which participants made the saccade into the wrong direction were counted as errors. Errors were detected when the participant’s eye gaze deviated more than 3° from the fixation point towards the wrong direction (in the pro-saccade task towards the placeholder on the opposite side; in the anti-saccade task towards the target). To ensure a sufficient number of trials with correct responses for the analysis of the saccade latencies, error trials were repeated at a later point of the experiment. Also, trials in which the criteria for a correct response were not met for other reasons (e.g., because participants failed to shift the eyes to the target area) or in which a saccade was recorded too early (<100 ms after target onset) were interrupted, and they were repeated at a later point of the experiment. When participants failed to respond correctly in a trial, written feedback on the display was provided, “Wrong or too slow”.

Twenty percent of the trials were catch trials in which no target was presented. In the catch trials, participants had to keep their eye gaze at display center. If they failed to maintain the eye gaze within the gaze contingent region at display center (within 3° of visual angle from display center along the horizontal axis), the corresponding catch trial was counted as an error, and it was repeated at a later point in the experiment.

#### Procedure

Each participant was tested in three blocks. First, the pro-saccade and the anti-saccade task were performed in separate blocks. Each of these two blocks consisted of 160 trials (80 supraliminal cue trials and 80 subliminal cue trials, in two separate mini-blocks). At the beginning of pro-saccade block and at the beginning of the anti-saccade block, participants were allowed to work through about 20 trials to practice the task. These practice trials were not analyzed. Block order of the first two blocks (and also of the two mini-blocks) was balanced across participants. The final block tested cue visibility. To make sure that participants understood their task correctly, prior to the cue visibility test block, participants were informed about the cue both verbally and in written form. They were informed that, in the supraliminal cue conditions, the cue would be a single white onset that would appear either to the left or to the right of fixation. They were now also informed that in the subliminal cue conditions one of the white onsets appeared as a cue, with a lead time of 16 ms before the other white onset on the opposite side. Participants were informed that they would have to indicate after each correct eye movement at which side the cue had appeared. The cue visibility test block consisted of four mini-blocks of 20 trials (block order of these mini blocks was the same as in the two main blocks). On each trial of the visibility test block, participants first had to perform a correct pro-saccade or a correct anti-saccade. Then, they were asked whether the cue had appeared on the left side or on the right side. To rule out that awareness-independent response activation by the cues contributed to the performance in the visibility test, a randomly varying stimulus-to-response mapping rule was applied (cf. Schoeberl et al. 2015). Participants had to discriminate cue locations by pressing the #2 and the #8 key on the number pad. Participants were informed about the actually pertaining stimulus-to-response mapping rule on a separate display that was presented right after the correct response to the target. There was no time restriction for the cue visibility rating.

### Results

#### Pro- and anti-saccade task

##### Saccade latencies

In each trial with a correct saccade, saccade latencies were measured as the time interval between the onset of target display and the time the onset of the saccade was detected (velocity threshold 30°/s, acceleration threshold 8000°/s/s). Saccade latencies that deviated by more than 2.5 SDs from the mean saccade latency in the corresponding condition were removed (2.9%). After this, the mean of the remaining data was calculated in each condition and the data were subjected to a repeated-measurements analysis of variance (ANOVA), with the variables task (pro-saccade task vs. anti-saccade task), visibility (supraliminal vs. subliminal), and validity (valid vs. invalid).

**Fig. 3:**
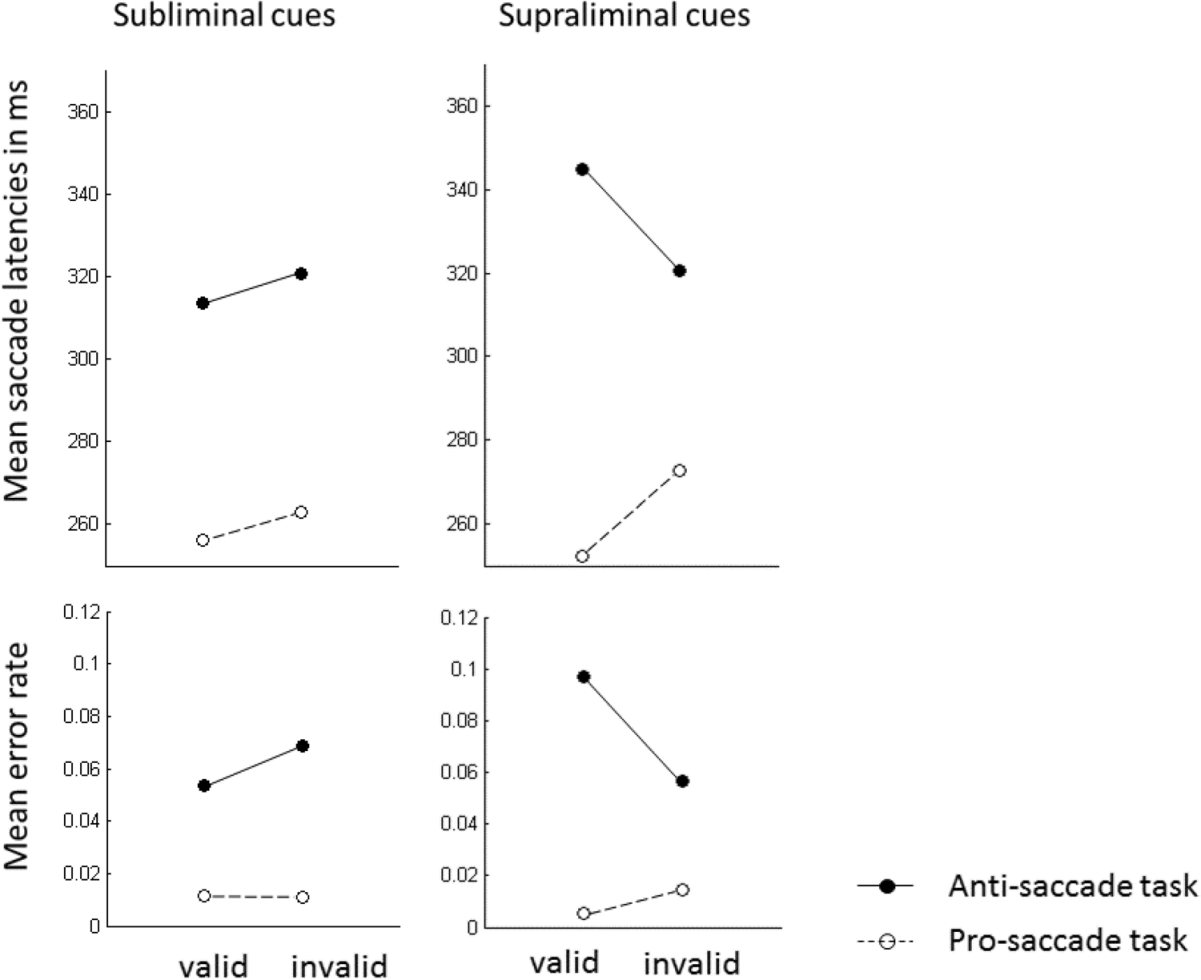
Mean saccade latencies and mean rate of errors in valid and invalid conditions for the pro-saccade task and for the anti-saccade task of *Experiment 1*. The *panels on the left side* correspond to the subliminal condition, and the *panels on the right side* correspond to the supraliminal cue condition

There were main effects for the variables task, *F*(1, 23) = 95.73, *p* < .001, $\eta_{\text{p}}^{2}$ = .81, and visibility, *F*(1, 23) = 5.50, *p* < .05, $\eta_{\text{p}}^{2}$ = .19. Saccade latencies were faster with pro-saccades (261 ms) compared to anti-saccades (325 ms), and they were faster with subliminal cues (288 ms) compared to supraliminal cues (297 ms). We observed a highly significant two-way interaction between the variables task and validity, *F*(1, 23) = 17.92, *p* < .01, $\eta_{\text{p}}^{2}$ = .44, and a highly significant three-way interaction between task, visibility, and validity, *F*(1, 23) = 11.71, *p* < .01, $\eta_{\text{p}}^{2}$ = .34. Because the three-way interaction was significant, we conducted two separate ANOVAs, with variables validity and task, once for the subliminal cue conditions and once for the supraliminal cue conditions. (1) In the ANOVA for supraliminal cue conditions, we found a main effect of task, *F*(1, 23) = 69.36, *p* < .001, $\eta_{\text{p}}^{2}$ = .75, and an interaction between task and validity, *F*(1, 23) = 17.33, *p* < .001, $\eta_{\text{p}}^{2}$ = .43. Post-hoc *t* tests showed that there was a cueing effect with faster responses in valid (252 ms) compared to invalid conditions (272 ms) in the pro-saccade task, *t*(23) = 5.01, *p* < .001, and a significantly reversed cueing effect with slower responses in valid (345 ms) compared to invalid conditions (321 ms) in the anti-saccade task, *t*(23) = 2.40, *p* < .05. (2) In the ANOVA for the subliminal cue conditions, we found a main effect of task, *F*(1, 23) = 79.44, *p* < .001, $\eta_{\text{p}}^{2}$ = .76, and a main effect of validity, *F*(1, 23) = 6.45, *p* < .05, $\eta_{\text{p}}^{2}$ = .22. The interaction between validity and task was not significant, *F*(1, 23) = 0.01, *p* = .93. Inspection of the data revealed that with subliminal cues the numerical cueing effect was 7 ms in the pro-saccade task as well as in the anti-saccade task. In the pro-saccade task mean saccade latencies were 256 ms in valid conditions and 263 ms in invalid conditions. In the anti-saccade task, mean saccade latencies were 313 ms in valid conditions and 320 ms in invalid conditions. Figure 3 shows the mean saccade latencies in each of the conditions.

#### Errors

When participants executed a saccade in the wrong direction, the trial was counted as an error. Errors occurred on average in 4.0% of the trials in which participants had to execute a saccade (1.1% in the pro-saccade task, 6.9% in the anti-saccade task). Other mistaken trials, in which participants produced a saccade too early (<100 ms after target onset) or in which participants missed the target area, occurred in 9.3% of the trials (7.2% in the pro-saccade task, 11.1% in the anti-saccade task). We computed the percentages of errors in each condition and performed arcsine-square root transformations. The transformed data were subjected to an ANOVA, with variables visibility, validity, and task, as above. The ANOVA^1^ showed a highly significant main effect for the variable task, *F*(1, 23) = 48.96, *p* < .001, $\eta_{\text{p}}^{2}$ = .68, confirming that participants were more prone to execute a wrong saccade in the anti-saccade task than in the pro-saccade task. There was also a three-way interaction between the variables task, validity, and visibility, *F*(1, 23) = 10.25, *p* < .001, $\eta_{\text{p}}^{2}$ = .31. We, therefore, conducted two ANOVAs, once for the subliminal cues and once for the supraliminal cues. (1) The ANOVA for the supraliminal cues led to a main effect of task, *F*(1, 23) = 53.09, *p* < .001, $\eta_{\text{p}}^{2}$ = .70, and to an interaction between the variables validity and task, *F*(1, 23) = 11.95, *p* < .01, $\eta_{\text{p}}^{2}$ = .34. Post-hoc *t* tests revealed a cueing effect with fewer errors in valid (0.5%) than invalid (1.5%) conditions in the pro-saccade task, *t*(23) = 1.82, one-tailed *p* < .05, and a reversed cueing effect, with more errors in valid (9.7%) compared to invalid (5.7%) conditions, in the anti-saccade task, *t*(23) = 2.64, *p* < .05. (2) The ANOVA for the subliminal cues had only a significant main effect of task, *F*(1, 23) = 32.02, *p* < .001, $\eta_{\text{p}}^{2}$ = .58. Other effects were not significant (non-significant *F*s < 1.04, non-significant *p*s > .32).

#### Errors in catch trials and correlation with the cueing effect

**Fig. 4:**
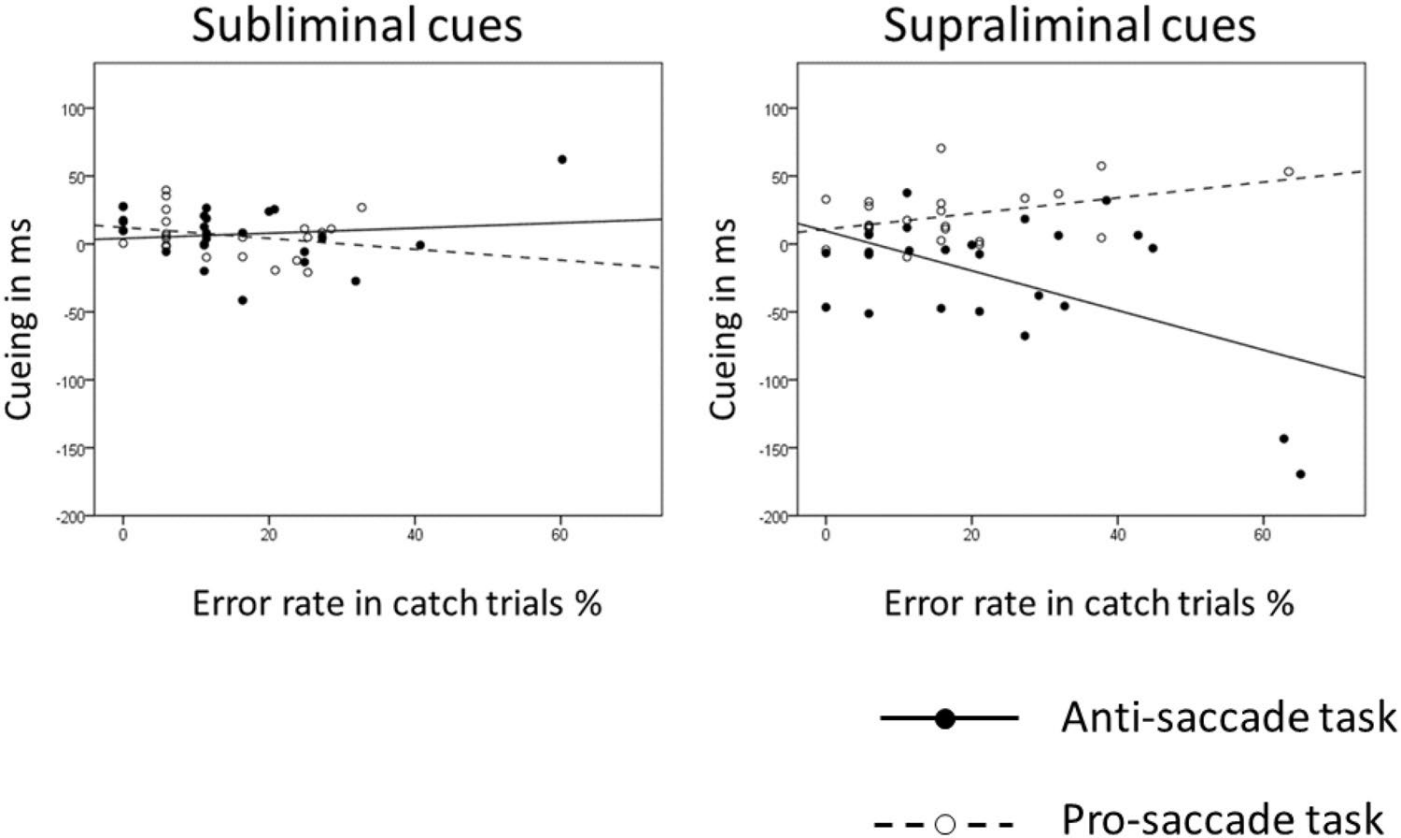
*Scatter plots* of the individual saccade latency cueing effects of Experiment 1 (invalid conditions minus valid conditions; on the ordinate) as a function of the error rate in the catch trials in the corresponding conditions (on the abscissa). The *plot on the left* shows the data for the subliminal cue condition. The *plot on the right* shows the data for the supraliminal cue condition. *Circles which are not filled* correspond to the data points for the pro-saccade task with the corresponding regression lines (*dotted lines*). *Filled circles* correspond to the data points for the anti-saccade task with the corresponding regression lines (*solid lines*). There was a significant positive correlation (*p* < .05) in the supraliminal cue condition with the pro-saccade task. There was a significant negative correlation (*p* < .01) with supraliminal cues in the anti-saccade task. Correlations in the subliminal cue conditions were not significant (non-significant *p*s > .22)

We counted the number of errors in the catch trials (i.e., the trials in which participants failed to maintain fixation at the display center throughout the trial) of each condition and correlated this value with the individual cueing effect in the saccade latencies of the corresponding condition. There was a significant correlation for supraliminal cues in the pro-saccade task (Pearson’s *r* = .42, *p* < .05) and a significantly negative correlation between the cueing effect for supraliminal cues in the anti-saccade task (*r* = −.53, *p* < .01). No correlations were found for the subliminal cues (in the pro-saccade task *r* = −.26, *p* = .22; in the anti-saccade task *r* = .13, *p* = .55). See Fig. 4.

#### Cue visibility

Cue visibility was assessed in a separate block at the end of the experiment. With the supraliminal cues, 11 participants had a hit rate (correct reports of cue location) of 100%. *d*′ was calculated for the remaining participants as the *z*-transformed probability of the hits (correct reports of cues at a location) minus the *z*-transformed probability of the false alarms (incorrect reports of cues at a location). *d*′ for the supraliminal cues (with 12 participants) was on average 2.9, and significantly different from zero, *t*(11) = 6.80, *p* < .001. One participant had 100% false alarms most probably due to a misunderstanding of the instructions of the cue-visibility task. With the subliminal cues, *d*′ was calculated as an average of all participants and was 0.10. This was not significantly different from zero, *t*(23) = 1.15, *p* = .26. We also computed binomial tests^2^ for each participant. None of the participants scored above the level of chance (*p* = .05). One participant scored significantly below the level of chance (*p* < .05)^2^.

### Discussion

Experiment 1 suggested that subliminal cues capture attention but do not directly activate saccades because the cueing effects did not drop with anti-saccades compared to pro-saccades. The pattern of results with the subliminal cues was in marked contrast to the control conditions with a supraliminal cue that showed a cueing effect in the pro-saccade task but a reversed cueing effect in the anti-saccade task. This pattern of results was much better in line with a saccade-activation effect of the supraliminal cues. Also the fact that the cueing effects were correlated with the errors in the catch trials with supraliminal, but not with subliminal cues, suggests that cue-directed saccade activation was a contributing factor in supraliminal conditions, but not in subliminal conditions. It is uncertain, however, whether cue visibility alone was the critical difference between the subliminal and the supraliminal conditions. Instead, the fact that a second abrupt onset appeared at the opposite side of the cue in the subliminal cue conditions, but not in the supraliminal cue conditions, created a striking physical dissimilarity between the two conditions and could have been responsible for the differences we observed in the subliminal and in the supraliminal cue conditions.

## Experiment 2

Experiment 2 was a control experiment which was conducted to find out if the difference in the anti-saccade task between subliminal and supraliminal cues could be attributed to the visibility of the cue or to physical differences of the experimental protocols. The latter possibility is likely because the onset of the cue in the supraliminal cue condition was not followed by a second onset at the opposite side of the display as it was the case in the subliminal cue condition. It is possible that the rapid succession of the two onsets in the subliminal cue conditions created a balanced signal in saccade motor maps with little or no lateralized activation by the cue and, therefore, little saccade activation in the direction of the cue (cf. Marino et al. 2012; Trappenberg et al. 2001). To test this contention, participants performed an anti-saccade task with visible cues in Experiment 2, but the second onset at the opposite side of the display was preserved: The supraliminal cue was presented in red so that it was clearly seen by virtue of its color difference to the white disk on the opposite side and the other items on the display. The second white disk appeared 16 ms after the cue and provided a second onset. If cue visibility alone accounted for the differences in the effects we observed in Experiment 1, Experiment 2 should yield negative cueing effects just like in the supraliminal cue conditions of Experiment 1. If the experimental protocol with two onsets at opposite locations was more important, we expected regular cueing effects like in the subliminal cue conditions of Experiment 1.

### Methods

#### Participants

Twelve new participants took part (*M*_Age_ = 21.5 years, SD = 2.1 years, 9 females).

#### Apparatus, stimuli, and sequence of events in a trial

The apparatus and the stimuli were the same as in the subliminal conditions of Experiment 1, with one exception. Only the cue was presented in red and was, therefore, visible by a color difference. It had the same brightness as the white onset at the opposite side (~14 cd/m^2^; *Yxy*-color coordinates 0.60, 0.34).

#### Task and Procedure

Task and Procedure were identical to the anti-saccade task in Experiment 1. After 20 practice trials, participants worked through 80 trials of an anti-saccade task. After that, participants worked through another 20 trials in which we verified that the colored cues were clearly visible by virtue of their color in a visibility test that was the same as in Experiment 1, with the exception that participants were now informed that the cue was always red so that they could locate the cue by its color.

### Results

#### Saccade latencies

Erroneous saccades (5.4%) and other mistakes (10.5%) were removed. Saccade latencies deviating by more than 2.5 SDs from the mean of the corresponding condition (2.9%) were removed, and the mean of each condition was computed after this step of data cleaning. The mean saccade latencies in the valid conditions and in the invalid cue conditions were subjected to a paired *t* test which did not show a significant result, *t*(11) = 1.13, *p* = .28, albeit the cueing effect being numerically positive with faster saccade latencies in the valid (351 ms) than in the invalid (361 ms) condition.

#### Errors

The percentage of erroneous saccades was computed per each condition and arcsine-square root transformations were applied. A *t* test comparing the transformed error rates revealed that errors were less frequent in valid (2.9%) conditions than invalid (7.9%) conditions, *t*(11) = 2.56, *p* < .05.

#### Cue visibility

Participants were very accurate in reporting the location of the red cue. Three participants had a perfect performance without a single error. Also the rest of the participants were very accurate. *d*′ of the remaining participants was on average 2.41 and differed significantly from zero, *t*(8) = 5.22, *p* < .001.

#### Correlation of the cueing effect with the error rate in catch trials

**Fig. 5:**
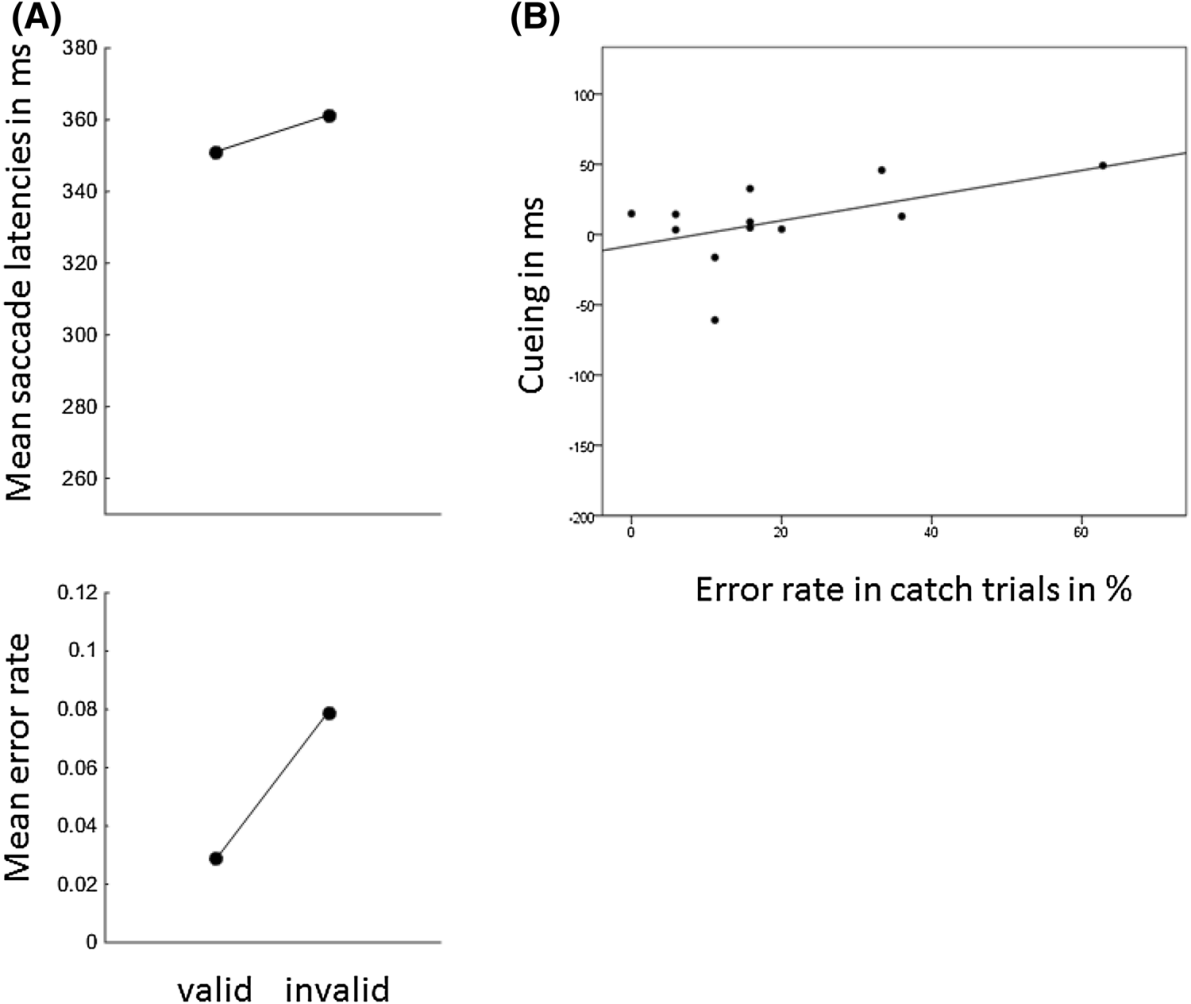
**a** Mean saccade latencies and mean rate of errors in Experiment 2. **b** *Scatter plot* of the individual cueing effects as a function of the error rate in the catch trials of Experiment 2. The correlation between the two variables was not significant (*p* = .074)

We counted the number of errors in catch trials and correlated the individual error rates with the saccade latency cueing effect in the same way as in Experiment 1 (see also Fig. 5). The correlation was not significant, although there was a trend for increasing cueing effects with increasing error rate (Pearson’s *r* = .53; *p* = .074). Notice that this was different from the supraliminal cue condition in Experiment 1, in which the cueing effect was negatively correlated with the error rate in catch trials of the anti-saccade task.

### Discussion

Experiment 2 showed that cue visibility cannot be held responsible for the different pattern of results that we observed between subliminal and supraliminal conditions of Experiment 1’s anti-saccade task. In Experiment 2, cueing effects in the anti-saccade task were positive despite the fact that participants clearly saw at which location the cue occurred. Although this result clearly shows that cue visibility was not the overall critical factor, it is on the other hand uncertain whether similar mechanisms were responsible for the positive cueing effects with anti-saccades in Experiment 2 and in the subliminal cue conditions of Experiment 1. In particular, the unique color of the cue in Experiment 2 could have enhanced its perceptual saliency and could, therefore, have boosted the cueing effect. To which extent this was the case remains unclear. We did not elaborate on this in more detail, however, because this was not the major objective of the present study.

## Experiment 3

Experiment 1 suggested that subliminal abrupt onsets did capture attention but had little direct saccade activation potential. It is possible, however, that effects of the subliminal cues on saccade programming were very short-lived and had disappeared at the time the required saccade was activated. In Experiment 3, we, therefore, used two different stimulus onset asynchronies (SOAs): In one block, Experiment 1’ results with subliminal cues were replicated with a pre-target cue. As the cueing effects in Experiment 1 were in general small, we slightly increased the pre-target SOA of the subliminal cues, a manipulation which might enhance the subliminal cueing effects (as the SOA arguably poses an upper boundary on the expected effect size; see Vorberg et al. 2003). We reasoned that larger overall cueing effects might increase the power of detecting the drop of cueing effects with anti-saccades compared to pro-saccades, if it were. That is, like in Experiment 1, we would expect cueing effects with pro- and with anti-saccades to be similar if cues captured attention. By contrast, we would expect the cueing effects to be reduced with anti-saccades compared to pro-saccades if cue-directed saccades were activated by the cues.

In addition, we tested in one block the impact of post-target cues on pro- and anti-saccades. In this block, cues appeared with an SOA of −50 ms following the target. In this condition, we expected the cues to be much less effective in terms of their attention capturing potential because the target had already been presented for 50 ms at the time of cue onset and location-specific target processing could in principle already have started. We, therefore, did not expect the attentional shifts to the targets to be influenced by the cues. If the cues only captured attention, we, therefore, expected the cues not to impact on the performance with the post-target SOA and we, therefore, also did not expect to see a cueing effect. Yet, effects of the cues on saccade activation could maybe be better observed if the cues appeared with a short delay after the target, especially with a short-lived cueing effect that would otherwise have dissipated at the time of target-saccade activation (cf. Buonocore and McIntosh 2008; Walker and Benson 2013). We, therefore, expected positive cueing effects with pro-saccades and a reversal of the cueing effects with anti-saccades if the subliminal post-target cues activated saccades just at the right moment in time.

### Methods

#### Participants

Twenty-four new participants took part (*M*_Age_ = 21.0 years, SD = 3.11 years, 17 female).

#### Apparatus, stimuli, and sequence of events in a trial

**Fig. 6:**
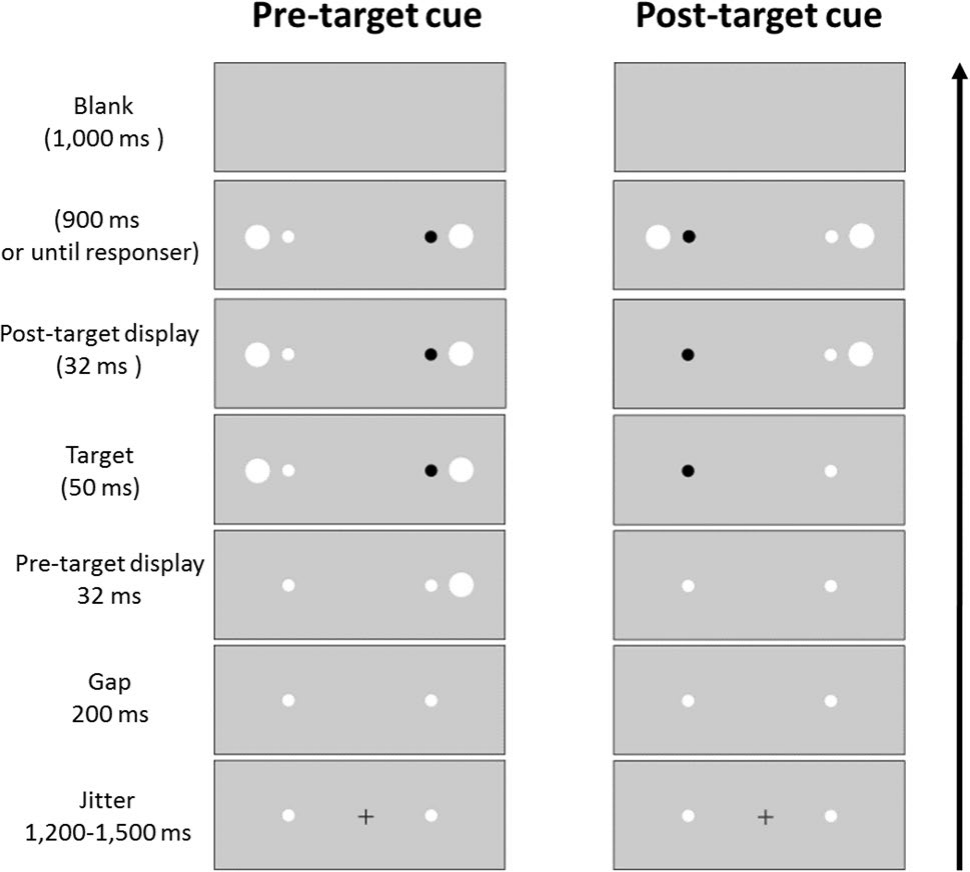
Sequence of events in Experiment 3. The *arrow on the right* indicates the flow of time from bottom to top. With a pre-target cue (*left column*), cues appeared 32 ms prior to the onset of the target and a second onset appeared at the same time as the target at the opposite side of the cue. With a post-target cue (*right column*), cues appeared 50 ms after the onset of the target and the second onset appeared after another 32 ms at the opposite side of the cue

The apparatus and the stimuli were the same as in Experiment 1. The sequence of events was similar to the subliminal cue conditions of Experiment 1, except for the SOA, see also Fig. 6. In one block, we presented the cue prior to the target like in Experiment 1, but the time interval was slightly increased (from 16 to 32 ms). The second cue-like white disk appeared at the same time as the target at the opposite side of the cue. In a second block, the cues appeared with a negative SOA of −50 ms following the target. In this condition, the second onset at the opposite side appeared 82 ms after the onset of the target so that the time interval between the two onsets was again 32 ms, as with the pre-target cues.

#### Participants

Participants worked through both blocks, once with a pro-saccade task and once with an anti-saccade task. In total, the main experiment consisted of four blocks of 80 trials each. Again 20% of the trials were catch trials in which no target appeared. At the end of the experiment, there was again a cue visibility test in which participants received another four mini-blocks of 20 trials each. Two of these blocks were with anti-saccades, two with pro-saccades. One block with each task was with pre-target cues, one block was with post-target cues. Block order was the same as in the main experiment. In each trial, participants were asked after they had executed a correct eye movement at which location the cue had appeared (left or right). The answer was given in the same way as in Experiment 1.

### Results

#### Saccade latencies

**Fig. 7:**
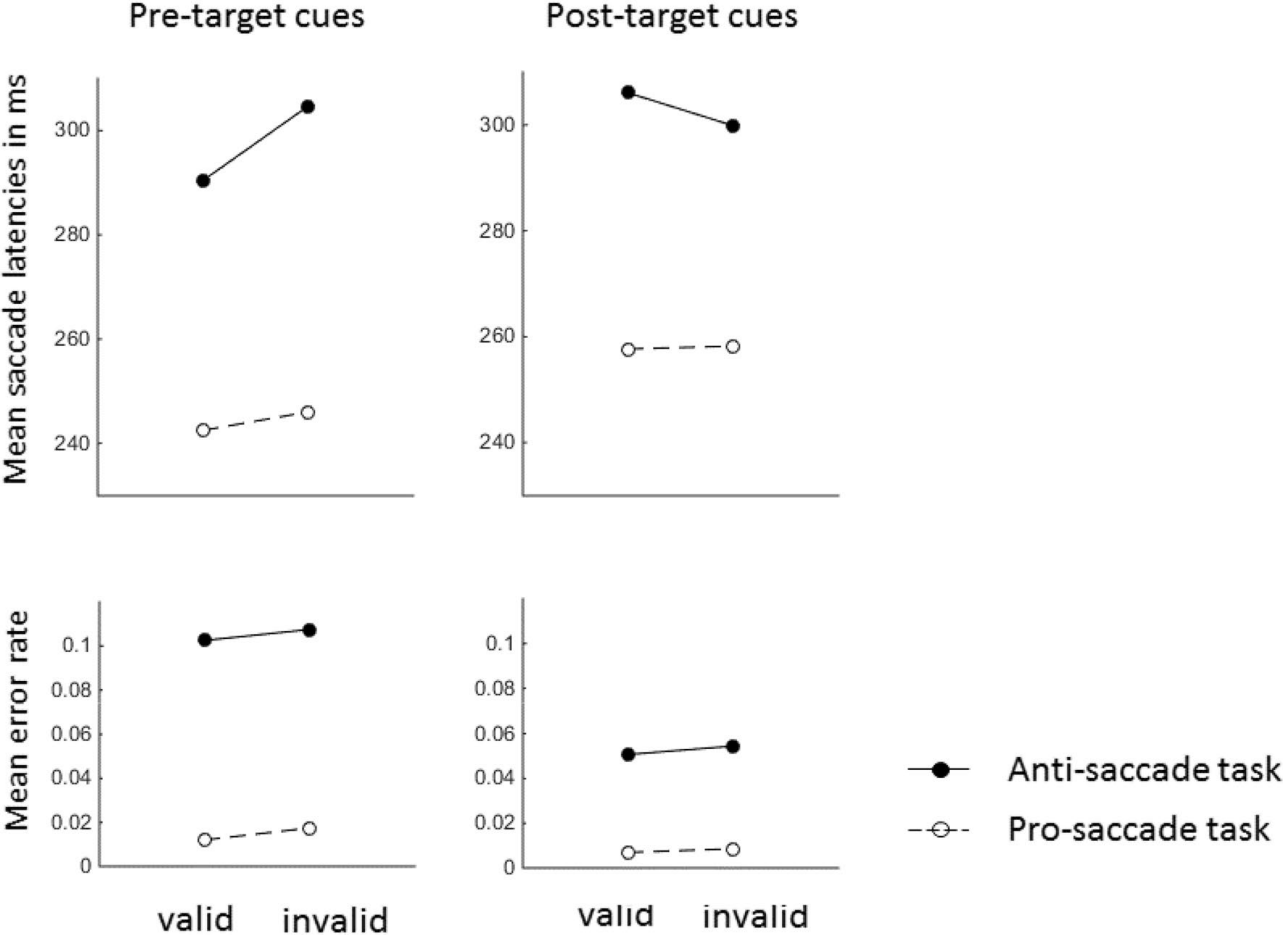
Mean saccade latencies and mean rate of errors in Experiment 3. The *panel on the left side* corresponds to the results in the pre-target SOA conditions. The *panel on the right side* depicts the results in the post-target SOA condition

For an overview of the results see Fig. 7. Erroneous saccades (4.5%), other mistakes (15.1%) and catch trials were removed. Saccade latencies deviating by more than 2.5 SDs from the mean of the corresponding condition (2.9%) were removed, and the mean of each condition was computed after this step of data cleaning. We fed these data into an ANOVA, with variables validity (valid vs. invalid), task (pro- vs. anti-saccade task), and SOA (pre-target vs. post-target). It led to a main effect of task, *F*(1, 23) = 90.90, *p* < .001, $\eta_{\text{p}}^{2}$ = .80, with faster pro-saccades (251 ms) than anti-saccades (300 ms). There was also a main effect of SOA, *F*(1, 23) = 6.82, *p* < .05, $\eta_{\text{p}}^{2}$ = .23, with faster responses with the pre-target SOA (270 ms) compared to the post-target SOA (280 ms). Importantly, there was an interaction between SOA and validity, *F*(1, 23) = 8.62, *p* < .01, $\eta_{\text{p}}^{2}$ = .27. With the pre-target SOA, there was a positive cueing effect, 9 ms, *t*(23) = 2.41, *p* < .05, but not with the post-target SOA, −3 ms, *t*(23) = 1.10, *p* = .29. The three-way interaction between all variables showed a trend but failed to reach significance, *F*(1, 23) = 3.65, *p* = .07, $\eta_{\text{p}}^{2}$ = .14.

Because the three-way interaction showed a trend, we conducted separate ANOVAs for the pre- and the post-target SOA conditions to check whether this trend could have reflected theoretically predicted effects. These analyses showed that a trend for the three-way interaction mainly reflected a tendency for a difference between the cueing effects in the pro-saccade task and the anti-saccade task with the pre-target SOA, *F*(1, 23) = 3.02, *p* = .10, $\eta_{\text{p}}^{2}$ = .12. But if anything, the cueing effect was larger with the anti-saccades, 14 ms, *t*(23) = 3.44, *p* < .01, compared to the pro-saccades, 4 ms, *t*(23) = .65, *p* = .52. Post-hoc inspection revealed that the absence of a statistically significant cueing effect in the pro-saccade task could be attributed to an outlier who had a large negative cueing effect in the pro-saccade task (−89 ms) which was more than two SDs away from the mean cueing effect in this condition. After removing this participant, the cueing effect with the pro-saccades was present, 8 ms, *t*(23) = 2.03, one-tailed *p* < .05, but cueing effects were still larger with anti-saccades (14 ms). With the post-target SOA, there were no reliable effects in the ANOVA except for the main effect of task. Numerically, the cueing effects were slightly reversed with the anti-saccades, but neither the main effect of validity nor the interaction between validity and task approached statistical significance (all non-significant *F*s < 1.5, all non-significant *p*s > .23).

#### Errors

The percentage of erroneous saccades was computed per each condition and arcsine-square root transformations were applied. The results were fed into an ANOVA as the above, with variables task, validity, and SOA. There was a main effect of task, *F*(1, 23) = 70.36, *p* < .001, $\eta_{\text{p}}^{2}$ = .75, with more errors in the anti-saccade task (7.9%) than in the pro-saccade task (1.1%). There was also a main effect of SOA, *F*(1, 23) = 11.58, *p* < .01, $\eta_{\text{p}}^{2}$ = .36, with more errors with the pre-target SOA (6.0%) compared to the post-target SOA (3.0%). There was a main effect of validity, *F*(1, 23) = 4.62, *p* < .05, $\eta_{\text{p}}^{2}$ = .17, with more errors in invalid (4.7%) compared to valid conditions (4.3%). In addition, there was an interaction between task and SOA, *F*(1, 23) = 6.64, *p* < .05, $\eta_{\text{p}}^{2}$ = .22, reflecting a bigger difference of the rates of erroneous saccades with the anti-saccade task between the two steps of the variable SOA (10.5 vs. 5.3%) compared to the pro-saccade task (1.5 vs. 0.8%). Other effects were not significant (all non-significant *F*s < .47; all non-significant *p*s > .50).

#### Cue visibility

We counted the number of correct reports of cue location and computed *d*′ in the same way as in Experiment 1. Overall, participants scored at chance level in the cue visibility test, *d*′ = .05, *t*(23) = 1.05, *p* = .32. Also, when cue visibility was separately assessed for the conditions with the pre-target SOA and for the conditions with the post-target SOA, overall cue detection did not significantly differ from the level of chance in either of these conditions (*d*s < .12, *p*s > .18). Only one participant scored above chance level when cue localization was separately assessed for each participant. When only the trials with pre-target cues were considered, three participants scored above chance level. Removing these participants from the ANOVAs on saccade latencies and erroneous saccades did not qualitatively change the results.

#### Correlation of the cueing effect with the error rate in catch trials

**Fig. 8:**
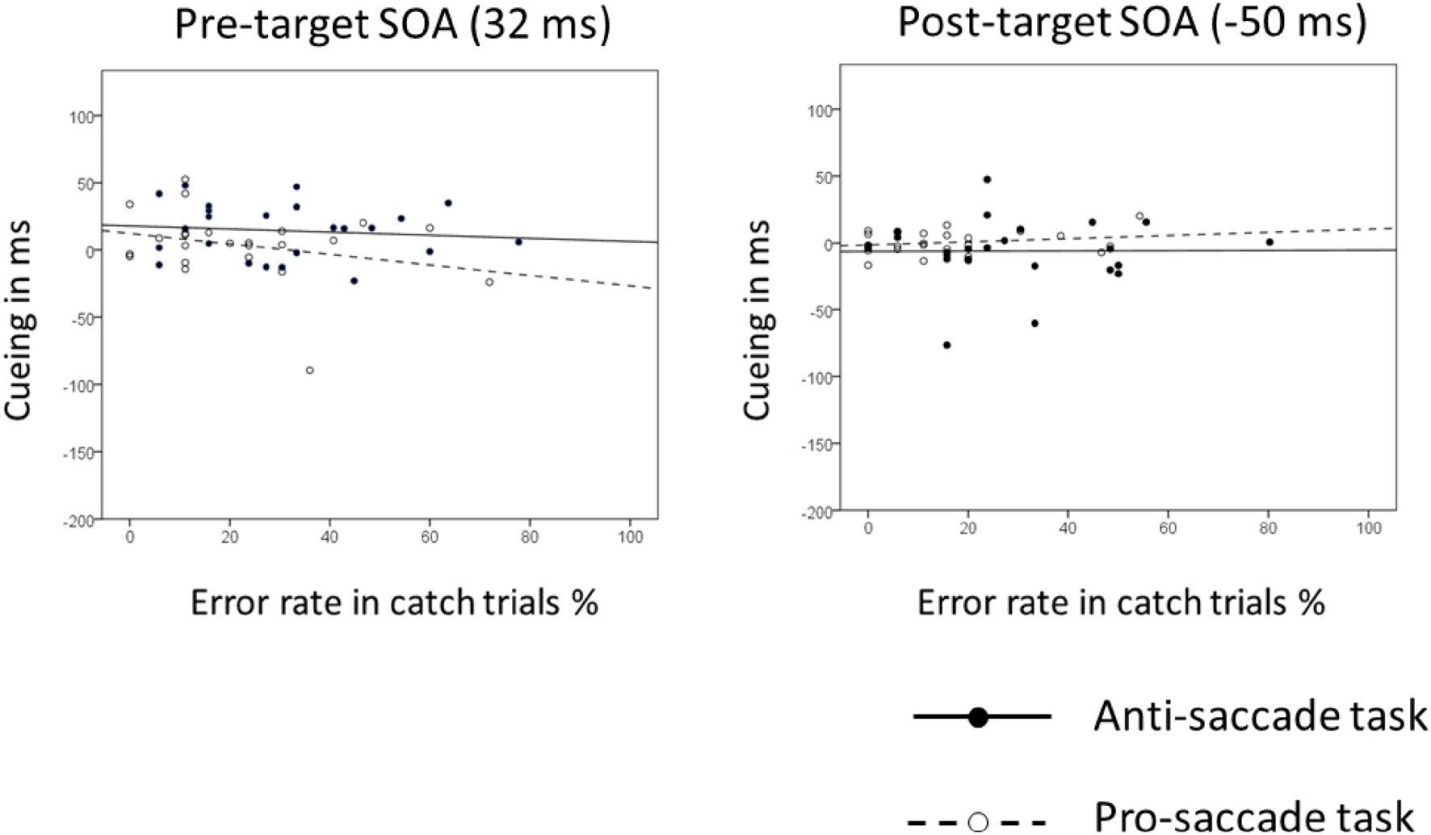
*Scatter plots* of the individual saccade latency cueing effects of Experiment 3 (invalid conditions minus valid conditions; on the ordinate) as a function of the error rate in the catch trials in the corresponding conditions (on the abscissa). The *plot on the left* shows the data for pre-target cue condition. The *plot on the right* shows the data for post-target cue condition. *Circles which are not filled* correspond to the data points for the pro-saccade task with the corresponding regression lines (*dotted lines*). *Filled circles* correspond to the data points for the anti-saccade task with the corresponding regression lines (*solid lines*). There were no correlations between the cueing effects and the error rates in this experiment (*p*s > .20)

Again we counted the number of errors in the catch trials and correlated it with the cueing effects in each of the conditions (see Fig. 8). None of the correlations was significant (*p*s > .20).

#### Discussion

Experiment 3 replicated the cueing effects with subliminal cues which we had observed in Experiment 1 with pre-target cues. There was a significant cueing effect with pre-target cues and again no evidence for a decrease of the cueing effect in the anti-saccade task compared to the pro-saccade task. If anything, the cueing effects tended to be larger and more robust in the anti-saccade task compared to the pro-saccade task.

In the post-target SOA conditions, we found no effects as we would have expected under the assumption that the cues only captured attention, without ever directly activating saccades. Notably, the cueing effect numerically reversed with the anti-saccades in the post-target SOA condition. Although this trend did not approach statistical significance, it could indicate a subtle impact of the cue on saccade programming. However, the reversal of the cueing effects with anti-saccades in the post-target SOA conditions could also be accommodated by the hypothesis that the cues captured attention because the anti-saccade task might have demanded participants to shift attention rapidly from the target location to the location of the saccade goal (e.g., Crawford et al. 2006; but see Klapetek et al. 2016), a process which might be facilitated by the capture of attention by a post-target cue at the location of the saccade goal. In sum, the results of Experiment 3 supported the conclusion that the subliminal cues mediated their effects on eye movements by attentional capture, not by direct saccade activation.

## General discussion

In the current study, we used pro- and anti-saccades to test to which extent the cueing effect by subliminal abrupt onset cues reflected attentional capture and to which extent the cueing effects reflected saccade activation towards the cue’s location. If saccade activation was involved, the cueing effects in the anti-saccade task should have been influenced by cue-directed saccade activation because only cues at invalid locations would have elicited the activation of the finally required saccade. Cues under valid conditions, by contrast, would have captured attention, but they would have activated the wrong saccade. In contrast, with the pro-saccade task, attentional capture and saccade activation would both have led to an increase of the cueing effect. The cueing effect was, therefore, expected to be less pronounced or maybe even reversed in the anti-saccade task compared to the pro-saccade if saccade activation by the cue was involved (cf. Laidlaw et al. 2016).

Most importantly, we found no indications for a drop of the cueing effects with anti-saccades compared to pro-saccades with subliminal cues—that is, the cueing effects with anti-saccades were the same (Experiment 1) or tended to be even larger (Experiment 3) with anti-saccades compared to pro-saccades. This result suggests that subliminal cues captured attention but did not directly activate saccades towards the cue location. The supraliminal cue condition of Experiment 1 confirmed that abrupt onsets did in principle have the potential to influence saccade activation in the way we had predicted. In the supraliminal cue conditions of Experiment 1, the abrupt onset at the opposite side of the cue was removed (Fig. 1 sequence of events in the middle). With this experimental protocol, there was a cueing effect in the pro-saccade task, but the cueing effect reversed in the anti-saccade task. In addition, there were correlations between the cueing effects and the error rate in the catch trials of Experiment 1. The more errors participants made in the catch trials, the larger were their cueing effects in the pro-saccade task and the smaller (more reversed) were their cueing effects in the anti-saccade task. This is in line with an explanation in terms of saccade activation because stronger saccade activation by the cues should lead to a higher error rate in catch trials, and this should increase the cueing effects in the pro-saccade task and decrease the cueing effects in the anti-saccade task. Experiment 2 suggested that cue visibility was not the decisive difference between the supraliminal and the subliminal conditions of Experiment 1. When cues were visible only by virtue of their color and a second onset at the opposite side was present, even supraliminal cues led to an advantage in valid compared to invalid anti-saccade task conditions. We, therefore, think that the experimental protocol, with onsets on both sides, was critical. It was, however, not the purpose of the present study to elaborate in more detail on the role of the second onset. The major objective of the present research was to study how subliminal abrupt onsets with the present experimental protocol influenced attention vs. saccades. We, therefore, replicated the major findings from Experiment 1 in Experiment 3 with a pre-target cue and tested how post-target cues that appeared after the onset of the target would impact on the cueing effects. The expectations were similar to those with the pre-target cues: if cues activated saccades directly, we expected a positive cueing effect in the pro-saccade task and a reversed cueing effect in the anti-saccade task. We expected not much of an impact by the cues if the effects were primarily attentional because search for the target could already have started when the post-target cue was presented. In line with the conclusion that cues attracted attention but had little or no potential to directly activate saccades, we observed no significant cueing effects with post-target cues in Experiment 3. Their cueing effect was numerically reversed with anti-saccades. But this tendency was not close to significance and could also be explained by attentional effects of the cues: An attentional cue appearing after the target could facilitate the reallocation of spatial attention from the target to the saccade goal which is sometimes assumed to be a necessary step in the execution of anti-saccades (e.g., Crawford et al. 2006).

Together, our results suggest that subliminal abrupt onsets impacted on saccades primarily by way of location-specific attentional processing, not by way of cue-directed saccade activation. What does this result mean with respect to the extant literature on the relationship between attentional capture and saccade preparation? According to the premotor theory of attention, shifts of visual attention are equivalent to the preparation of eye movements towards the attended locations (Rizzolatti et al. 1987, 1994; Sheliga et al. 1994, 1995). This means that shifts of visual attention, even when they are covert (i.e., not accompanied by an actual movement of the eyes towards the attended locations), are always accompanied by the preparation of a saccade. The generality of this claim has been called into question earlier especially because endogenous orienting can seemingly be dissociated from saccade activation in certain conditions (Belopolsky and Theeuwes 2012; Smith and Schenk 2012). But it is less clear to which extent exogenous attention can be dissociated from saccade activation (Belopolsky and Theeuwes 2012; Craighero et al. 2001; Smith et al. 2004; Smith and Schenk 2012; but see MacLean et al. 2015). The present data suggest that it might be possible to dissociate exogenous covert attention from saccade activation under certain conditions because the subliminal cues did seemingly capture visual attention without at the same time influencing saccade programming directly. However, there is a caveat to this conclusion: The time course of attentional effects and direct oculomotor effects of subliminal cues might differ and the effects of the cues on the oculomotor system might be much more short-lived and/or operate at different time scales than their attentional counterparts. As a consequence, attentional effects could have been isolated in the present study simply because the effects of the cues on the oculomotor system were transient and had already disappeared at the point in time when the saccades were programmed and executed. This might especially be a concern in the anti-saccade task where response latencies were larger than with pro-saccades. However, the fact that we failed at detecting effects of subliminal cues with a post-target SOA in Experiment 3 casts some doubt on this explanation. If the cues led to saccade activation at any point in time, the post-target cues would have had more of a chance to directly influence saccades, and yet even the post-target cues failed to show differences of the cueing effect depending on whether a pro- or an anti-saccade task was required. In sum, the present data suggest that the direct saccade activation potential of subliminal abrupt onset cues is very limited relative to their potential to capture visual attention although transient activation of saccades cannot be ruled out.

## Footnotes

1 A separate ANOVA was conducted with the rates of mistakes (trials in which participants failed to maintain gaze contingent fixation before the target appeared and trials in which they missed the target area). Besides a main effect for the variable task, *F*(1, 23) = 6.21, *p* < .05, $\eta_{\text{p}}^{2}$ = .68, with again more errors in the pro-saccade than in the anti-saccade conditions, it showed no significant effects (non-significant *p*s > .15, non-significant *F*s < 2.21).

2 We checked whether excluding the participant whose score in the binomial tests differed significantly from chance influenced the results. The ANOVAs on saccade latencies and erroneous saccades were repeated but none of the results changed qualitatively.
